# High-tech biomedical research: lessons from Iran's experience

**DOI:** 10.1186/1475-925X-7-17

**Published:** 2008-05-23

**Authors:** Ali Samadikuchaksaraei, Kazem Mousavizadeh

**Affiliations:** 1 Department of Biotechnology, Cellular and Molecular Research Center, Faculty of Allied Medicine, Iran University of Medical Sciences, Tehran, Iran; 2 Cellular and Molecular Research Center and History of Medicine, Islamic & Complementary Medicine Research Institute, Iran University of Medical Sciences, Tehran, Iran

## Abstract

Iran has recently made a significant progress in the field of biomedical science and launched an appreciable number of new high-tech biomedical research projects. Review of Iran's experience in advancing its biomedical research and the pitfalls the country encountered during the years of its progress could be of interest to other countries with similar technological conditions. As needs assessment and human resources have pivotal roles in any research infrastructure, here, we have delineated these factors and explored ways by which optimum advantage could be gained from them.

## Background

Biomedical research activities in Iran have significantly increased in recent years. Several factors contribute to such improvement. For example, research budget allocated to the health sector that was 5% of total public funding in research in 1997[1] was increased to 9% in 2005[2]. Additionally, employment of faculty members for health research and education that was 9 783 faculties in 1999[1] was increased to 11 050 in 2002[2]. Furthermore, 4 biomedical research centers throughout the country in 1997 have been increased to 64 in 2006[3]. Moreover, the number of Iranian articles indexed under PubMed showing 19 times increase from 1997 through 2006 is another sign of a forward movement.

These developments are the results of highly coordinated efforts of Iranian biomedical research community. This community is expanding its research capabilities and activities over a range of subjects, ranging from epidemiological studies to high-tech medical interventions, to address the national health problems. Iranian scientists have succeeded to set up numerous high-tech research centers and start projects in technologically advanced fields including stem cell biology and cell therapy, tissue engineering, monoclonal antibody, recombinant drugs and proteins, radiobiology, and nanobiotechnology.

The high-tech Iranian research initiative is intended to build a strong national infrastructure to assist country's development in health sector. Any new initiative might face potential pitfalls and inherent obstacles. Therefore, it would be valuable to review the current impediments to the progress of high-tech biomedical research in Iranian setting and consider such drawbacks for troubleshooting purposes. For careful assessment of such progress, it is important to consider fundamental factors upon which Iran's new high-tech biomedical research is developing.

Amongst several components that constitute the research infrastructure, two main areas in Iran's research infrastructure need particular attention. These areas include: 1) proper and objective-based assessment of societal needs and bringing these needs to the attention of policy makers to receive relevant support, and 2) creation of an environment in which research is considered equally important as provision of service. The latter highly affects the quantity and quality of activities of human resources in a research system. Accordingly, here, we review our experience with regards to needs assessment and human resources. Awareness of our experience in Iran will be useful for other developing countries because states with similar technology situations have many overlapping issues.

## Needs assessment

Determination of research priorities is a subject of paramount importance, particularly when limited resources are available. Although systematic and evidence-based assessment of needs is performed in Iran and international collaborations have been established, eg. with the Council on Health Research for Development (COHRED)[4], for robust determination of research priorities, still most priorities are determined by subjective opinion of the experts in the field, which inevitably involves subjective bias. The priorities determined by subjective assessment will not attract the attention and support of policy makers as strong as the priorities determined objectively.

An example of objective-based needs assessment is the effort of Iranian nutrition scientists to provided sufficient evidence [5-7] for convincing the government that the country is suffering from several nutritional deficiencies and requires systematic strengthening of the national nutrition capacity. As human resource is a major player for biomedical research infrastructure, the government decided to start the strengthening by setting up a comprehensive multidisciplinary nutrition postgraduate program (at both MSc and PhD levels) with a view to the proposed cell-to-society model. This program aims to train experts who address a broad range of nutritional issues from molecular to political level. To achieve this goal, several postgraduate degrees in the fields of cellular and molecular nutrition, nutrition epidemiology, community nutrition, and food policy and nutrition intervention were proposed for this program. To design these degree programs, a major international consultancy was conducted over 19 months that included more than 2000 consultancy-hours with 48 scientists from 7 countries. The consultancy was funded by a cooperative agreement between the Iranian government and the World Bank.

This was just the planning phase of the human resources strengthening part of the program. It could be imagined what the rest of program will be. For example, the high-tech part of the program that includes cellular and molecular nutrition will require heavy investment on equipment. This was agreed by policy makers and will be supported by funding bodies because of the suitable environment created by this initiative.

The other example of a successful objective-based need assessment is identification of need for systematic study of Iranian medicinal herbs, using cutting edge technology. The world market for herbal medicines based on traditional knowledge is now estimated at 60 billion dollars[8]. Iran is home to 80% of the world's known medicinal herbs, however Iran's annual export only amounts to 10 million dollars of raw material. The potential of increasing this amount up to 500–600 million dollars of processed material exists[9]. Therefore, a coordinated effort has started to take advantage of the valuable pool of Iranian herbal plants. Regulations were set for herbal medicine[10] and a number of high-tech research centers established throughout the country to extract, characterize, and formulate the active components of these herbs.

These successful achievements were the result of proper evidence-based assessment of needs and appropriate presentation of those needs to policy makers. Accordingly, it is recommended that scientists in developing countries pay more attention to and emphasize more on local evidence-based needs assessments, rather than subjective experts' opinions or international priorities. This may necessitate short- or long-term training courses to teach needs assessment methodologies to scientists who are in charge of determining national research priorities.

Local evidence-based assessment of needs equip scientists with tools that are more convincing to local policy makers and funding bodies. An attitude study performed by enrollment of nationals of five developing countries (China, Thailand, India, Egypt and Kenya) has shown that local audience are more convinced by the results of a research performed and published locally, compared to internationally performed research[11].

## Human resources

"Service" and "research" are two main tools that can identify and be used to solve societal problems. Therefore, these tools should be equally valued and dealt with. One of the basic rules in setting up a successful research system is taking advantage of people who believe on the importance of research for solving societal problems. These people can be created in an environment that promotes this belief by considering the factors discussed below.

### Salaries

Scientists are the main engines of any research machinery and their incentive and endurance are essential for setting up and continuation of research activities within different fields. Therefore, special attention should be paid to provide scientists with requirements enabling them to perform robust researches. This issue has been frequently noticed by others[12] and recognized as one of the factors contributing to the brain drain phenomenon[13].

In comparison to developed countries, performing research is harder for scientists in developing countries. Among many reasons, scientists in developing countries struggle more for provision of proper set-up and research funding. Therefore, it seems that scientists in developing countries need more encouragement and incentives, compared with scientists in developed countries.

Usually, researchers' income is the foremost issue discussed when scientists' conditions are studied by any reviewer[14]. The present review concurs with the importance of income in retaining scientists in the research line. In Iran, many scientists have to engage in side and private activities to be able to live in conditions comparable with other equally educated individuals totally involved in non-research activities.

High engagement in non-research activities may decrease the time and diminish the enthusiasm scientists devote to their research. Many academics prefer professional activities that are more rewarded financially. For example, in addition to their salary, academics in teaching hospitals receive extra-payment for the professional services they provide during routine working time. Such financial incentive can by far supersede minimal incentives scientists may receive through a research grant application. In case of clinicians, the major source of income is through patients they visit. Therefore, most clinicians are inclined more towards visiting high number of patients rather than conducting scientific activities with no or minimal financial incentives.

An assessment of 186 academics of Gilan University of Medical Sciences (Iran) using the BARRIERS scale originally developed by Funk *et al*[15], revealed that about 70% of theses academics believe that low income is one of the constrains for their research activities[16]. On the other hand, promotion from assistant to associate and full professor only increases the income by 15% and 17% respectively[17]. A similar situation has been reported from Saudi Arabia[18].

Generally speaking, as activities with higher return on investment are preferred, and because investment of time on routine professionalism in a private setting leads to higher financial income in comparison with investment of time on setting up and running an advanced hard-reaching biomedical activity, the former is preferred by most individuals.

One of the best ways to encourage involvement in high-tech research activities is to increase research wages to the level comparable to that of other professional activities. This change should be so dramatic that lead to increased involvement of current scientists and attract "talented" students to choose a scientific career upon their graduation. Also, there should be higher wages for the research activities that need a labor-intensive set up. In addition, in accordance to Aziz *et al*[18], we believe that the salary gap between the academic positions (assistant, associate, and full professor) should be increased by 100%–200% or even more to make academic promotion an economically profitable milestone in developing countries.

### Teaching versus research

Giving strong priority to teaching rather than research is another policy in the higher education system, which is believed to be counterproductive[16]. The number of staff in each academic department is mainly determined by the teaching or professional service of that department, rather than the load of research projects. Moreover, extra-payment an academic receives for additional teaching hours is often higher than what could be obtained as the results of a research project. Therefore, many academic staff prefer extra-teaching hours more than research. Teaching overload has also been noted by academics of other countries[19].

Decreasing the teaching responsibilities by employment of more academic staff and making the research wages higher than teaching wages should be considered as one of the major milestones in the research development policy.

### High-tech versus low-tech

Considering time limitation of scientists along with the labor-intensive setting up of advanced experimental research, the scientists are usually pushed into areas such as epidemiology that require less staff and effort[20]. Accordingly, it is anticipated that the output of low-tech studies can be more than high-tech ones. But, to produce a visible health effect, epidemiologic studies should be complemented by other studies, usually at an advanced high-tech level. Unfortunately, the high-tech studies that complement epidemiologic studies are infrequent in Iran and other countries with similar technology situation[21,22]. For example, an unpublished survey of Iranian biomedical publications performed by one of the authors in 2005 through PubMed and IranMedex (an online index of Persian medical articles) showed that only 0.4% of nutrition-related papers included genomics, whereas 43.7% of those studies included fields such as epidemiology, education, economy, food policy, food health and nutrition assessment.

To increase the incentive for faculty members to conduct high-tech studies, it would be useful to consider more score for high-tech research in metrics used for academic promotion. Also, wages for high-tech biomedical research should be higher than low-tech research.

### Research habit

Establishing the habit of research in academia is a very fundamental issue that should be properly addressed. Low interest in research activities is a matter reported not only for Iran[16] but also for other culturally similar countries[23,24]. This low interest is a multifactorial phenomenon. But, we believe that one of the deeply-rooted reasons that negatively affects the training of graduate students in conducting research is the significant barrier provided by extremely competitive entry exams required for enrolling in higher education in Iranian universities.

The students have to "win" the competitive entrance exams for entering into most undergraduate and postgraduate degree programs, and even postgraduate professional training programs. Such condition is particularly more pronounced in programs whereby higher income is expected upon graduation. To pass these exams, applicants need to learn several voluminous textbooks. This needs heavy study schedules, which is usually complemented by enrollment in private entrance exam preparation classes. Such entrance exam preparation usually starts from the third grade of high school and continues until the person is accepted into his desired program.

The entrance exams are in form of multiple choice questions (MCQ). A student who wishes to enter a PhD program has to win at least 3 national entrance exams with MCQ formats (BSc, MSc and PhD entrance examinations). For being successful in all three entrance exams, the students usually undergo an intensive 8-year MCQ-oriented training in addition to the conventional training they receive for their degree (usually the applicants prepare 2 years for BSc entrance exam, 4 years for MSc and 2 years for PhD entrance exams). These 8 years of intensive training makes the "winning" students robust MCQ answering professionals. The PhD students who are the "champions" of these competitions are the best tutors for the private entrance exam preparation courses. So, most of them become involved in tutoring applicants as a high-paid part-time job. The PhD program has the huge task of turning these examination-minded students into critically thinking individuals. This is a very hard task in the above context and its failure rate is noticeable.

Team working, communication, writing and research management skills are other abilities that are not well developed in the training of those students. For example, in a 2004 report of assessment of research managers of 39 Iranian universities of medical sciences, it was found that 40% of research managers did not possess adequate research management skills[25]. Critical thinking, reviewing the literature, and research managements are important tools that a scientist needs to acquire. However, those skills can be acquired only through long-term training, which should be preferably started when the students are more flexible, i.e. during the undergraduate program. To be effective, student should develop those skills and consider them as important components of research. A somewhat similar concern was also expressed by Saudi scientists[18].

The best way to counteract the examination-oriented thinking is to remove the competitive entrance exams for application to postgraduate programs. In accordance with many high-ranking universities worldwide, the postgraduate applicants can be selected based on their curriculum vitae and the letters of reference. Training of research skills can be started during the undergraduate program by a mandatory short-term (e.g. summertime) research internship carried out by the student as a member of a research team. Likewise, the undergraduate teaching method should be optimized for development of students' abilities such as critical thinking, writing, management and communication skills.

### Communication

As mentioned, communication skills are among fundamental requirements that a scientist should possess. Scientists should be substantially trained to acquire strong communication skills with emphasis to communicate with policy makers. This will help bringing science to the level of decision-making. For example, upon analysis of the views of a group of Mexican researchers and policy-makers, it was recommended that training of both groups is needed. The researchers need to know how to communicate with policy-makers, and policy-makers need to consider how they imply the results of research[26].

A good example of proper dialogue between scientists and policy-makers is the discussion between then president emeritus of the University of Texas M. D. Anderson Cancer Center and the Cuban president. The discussion was taken place in 1980 on the possibility of positive effects of leukocyte interferon on malignant diseases and followed by the success of Cuban scientists in isolating this protein from human blood. This encouraged the Cuban government to properly invest on the biotechnology sector[27] in a way that Cuba is now exporting recombinant biopharmaceutical products to more than 30 countries[28].

To properly develop highly critical skill of communication, students should be trained in different courses by long-term and continuous assignments. The training should start from undergraduate period and continued to the postgraduate level.

### Collaboration

Collaboration with other research institutes and conducting joint projects is a basic step in building capacity for scientific research[29]. Most high-tech biomedical research projects need integrated multidisciplinary approaches and exchange of ideas among scientists with different expertise. Joint projects involving several centers are encouraged by the Iranian government and the research centers are awarded higher scores in their annual evaluation for joint multi-institutional projects[30] However, this matter is not adequately encouraged by academic position promotion regulations. For promotion purposes, academic staff receive highest score for single-author publications. Increase in the number of authors per article decreases the promotion score[17]. The same regulation that exists in Saudi Arabia[31] has negatively affected conducting research in a way that most scientists tend to decrease their number of collaborations for a single project. This is a highly sensitive issue that should be properly addressed and promotion regulations be revised to make collaboration a promotion-friendly event.

### Professional-scientists

The final concern in regards to biomedical scientists is the extent of their exposure to the health concerns in the real situations[12,32]. Most biomedical scientists just follow their curiosity, independent of societal needs, and are more concerned about their publication records than the application of their research output. Two models have been proposed to address this problem: increasing the communication between scientists and health professionals, and training professional-scientists. The value of professional-scientists is not always well appreciated. A professional-scientist such as a physician-scientist, pharmacist-scientist, or dentist-scientist has received formal research training usually leading to a PhD degree. As a result, such individual is more capable to bridge the gap between professionals and basic science technologies[33].

To justify the existence of physician-scientists, the "paralyzed academic investigator's disease" (PAID) syndrome has been described in 1986 after analysis of more than 1000 cases[34] and was widely accepted by the medical community. PAID syndrome manifests itself when a brilliant investigator uncovers a very important finding, while lack of knowledge in basic or clinical fields does not allow the scientist to translate the findings into proper application. However, since many scientists with major discoveries become renowned in their field, other members of the medical community do not notice that the lack of application of the discovery was potentially due to lack of knowledge or faulty design of the action plan. As the result, there would be unnecessary impediment of the progress of a potentially feasible and important application along with discrete use of time and financial resources.

The current setting of the Iranian health system requires the professional-scientists to concentrate mostly on academic and non-professional activities. Most physicians who pursue a research training program leading to a PhD will not follow available specialty training programs. This is inconsistent with the philosophy of training professional-scientists. Recently, this problem was identified by the Undersecretariat of Education in Iranian Ministry of Health and Medical Education. The Undersecretary is currently passing new bylaws to facilitate training of professional-scientists. The current regulations should be changed to encourage scientists to receive a specialized professional training and qualification and the professionals to receive a research training and degree. The professional-scientists should be involved in both professional and scientific activities to better fill the gap between extreme professionalism and extreme science.

## Conclusion

In conclusion, it should be pointed out that in developing countries the high cost of high-tech biomedical research necessitates investment by government. This investment should be properly made in fields needed for country's development. The needs should be defined, justified and approached by local scientists who are research-oriented and capable of establishing proper dialogue with policy makers and funding bodies. As the country's development is promoted by science, current policies should be optimized to make "science" a profitable activity and this optimization should be performed by scientists themselves, keeping in mind that robust science is made by robust scientists.

## Competing interests

The authors declare that they have no competing interests.

## Authors' contributions

AS conception, drafting and final approval of the manuscript, KM drafting and final approval of the manuscript.
